# The four abilities of emotional intelligence as predictors of health risk behaviour: what role do impulsivity and sensitivity to reward play in this relationship?

**DOI:** 10.1192/j.eurpsy.2024.498

**Published:** 2024-08-27

**Authors:** M. T. Sanchez-Lopez, P. Fernández-Berrocal, R. Gómez-Leal, M. J. Gutiérrez-Cobo, R. Cabello, A. Megías-Robles

**Affiliations:** ^1^Department of Basic Psychology; ^2^Department of Developmental Psychology, University of Malaga, Málaga, Spain

## Abstract

**Introduction:**

Risky sexual relationships, reckless driving or initiating drug use are examples of health-related risk behaviours that are often related to poor emotional abilities (emotional identification, emotional understanding, facilitating thought and emotional regulation). However, the mechanisms by which this relationship operates have been relatively little studied. It is well known that certain personality traits such as impulsivity and sensitivity to reward are strongly related to risk-taking behaviour.

**Objectives:**

The aim of this work was to explore the role of these two traits in the relationship between each of the different abilities/ branches of emotional intelligence and health risk behaviour, as well as to identify the emotional ability that best predicts this relationship.

**Methods:**

A community sample of 250 participants (Mage = 23.60; 72% women) was used to measure levels of emotional intelligence in each of its branches (through the performance-based ability test MSCEIT), and levels of health risk behaviour, impulsivity and sensitivity to reward.

**Results:**

The results supported the existence of a negative relationship between the four emotional abilities and health risk-taking. Mediation analyses that included all four MSCEIT branches as predictors revealed an indirect effect of the “managing” branch on risk-taking, being the most important branch in predicting health-related risk-taking, due to its effects through impulsivity and sensitivity to reward.

**Conclusions:**

Our results suggest that a strong negative relationship exists between emotional management ability and health risk-taking, highlighting that the emotional components of impulsivity and levels of sensitivity to reward have been shown to be among the mediating factors underlying this relationship. Further experimental research is needed to confirm the role of emotional intelligence, and in particular emotional management, as a protective factor for risk-taking behaviour.

**Disclosure of Interest:**

None Declared

